# A House Divided against Itself

**Published:** 1896-12-26

**Authors:** 


### The Hospital

A Journal of

The Medical Sciences and Hospital Administration.

Vol. XXI.—No. 535.] [Dec. 26, 1896.

### A House Divided Against Itself.

Until the decision of the Divisional Court, which is the Court of Appeal for reviewing the decisions of inferior courts, was given last week in the case of Breay v. Browne, we were precluded from commenting upon what passed at the trial of that action before Mr. Commissioner Kerr and a County Court jury on October 5th last, as well as upon the facts which led to that action being brought. And, although the action was, from many points of view, an important one, we did not think that it was of such interest as to warrant us in inflicting upon our readers a *verbatim* report of counsel's speeches, or the evidence of the various witnesses. Moreover, the space at our command was, as usual, limited. The idle and curious, therefore, are referred to the *Nursing Record* of October 17th, as our contemporary obligingly devoted the whole of that enlarged issue to a *verbatim* report of what it believed would be "hereafter regarded as an historical event in the Nursing World."

But the Divisional Court (consisting of Mr. Justice Wills and Mr. Justice Wright) having finally decided the matter by refusing, for reasons to which we shall presently refer, to grant Miss Breay leave to carry the case to the Court of Appeal, it becomes our bounden duty to keep silence no longer. The best interests of the nursing profession demand that we should speak, and speak plainly.

In order, however, to properly appreciate the judgment of the Divisional Court it is necessary to state shortly what were the facts which led to this miserable and contemptible dispute. In substance they are as follows. For some time past a small and turbulent section of the members of the Royal British Nurses' Association has expressed dissatisfaction at the manner in which the affairs of the association were being conducted. Considering the falling off in the number of its members and the consequent diminution of its income, we are not surprised; but this is, of course, by the way.

One of the bye-laws of the association provides that, "No resolution shall be proposed at any annual or general meeting unless the full text of the resolution shall have been sent in writing, and by registered letter, to the secretary at least three weeks previously for insertion upon the agenda of the said meeting." On June 30th last Miss Breay wrote out the full text of a resolution condemnatory of the management of the R.B.N.A., which she desired to move at its annual meeting, which had been announced to be held at St. Bartholomew's Hospital on July 22nd. The letter containing that resolution she handed in at the Vere Street Post Office, and in exchange for the sum of 3d., which she then paid, was given a receipt upon which the word "express" was written in pencil, none of the blanks the receipt-form contained being filled up as is usual and customary.

The full text of the resolution was inserted upon the agenda of the meeting. This, we understand, was done at the instance of the Executive Committee in order that, whoever presided at the meeting might decide whether, or not, the bye-law in question had been complied with. The meeting was duly held on July 22nd, Sir James Crichton Browne presiding. Sir James Crichton Browne had not intended to be present, but, at the request of H.R.H. the President, he came up from Scotland to take the chair, arriving in London about two hours before the meeting; and, until he entered the hall where the meeting was held, had never seen Miss Breay nor heard of her or her resolution. He was then informed that Miss Breay's resolution had not been received in a registered letter as required by the bye-law to which we have already referred.

When the resolution was reached Mr. Fardon (the secretary) rose, announced that it had not been received in a registered letter, and requested Sir James Crichton Browne to decide whether it was, or was not, in order. After referring to the bye-law in question, Sir James Crichton Browne intimated that he was of opinion that the resolution was not in order. He was then shown the envelope in which it had been sent, and found, as the fact was, that that envelope lacked the indicia of a "registered" letter in that it was not crossed in blue, that it bore no stamp, and was not marked with the letter "R" or the word "Registered." Sir James Crichton Browne thereupon came to the conclusion that there had been a blunder somewhere, but, being of opinion that he was bound to adhere to the strict words of the charter and bye-laws of the Association, he ruled that the resolution was out of order.

Miss Breay immediately placed the matter in the hands of her solicitor, whose chief desire seems to have been to express regret that an action was inevitable.

He was at once informed by Sir James Crichton Browne's legal adviser that the Executive Committee were, as they always had been, desirous that Miss Breay's motion should be brought on and dealt with, as the Executive Committee were fully prepared to meet the charges she had made against them, and it was pointed out that a special general meeting could be called to enable Miss Breay—if she so wished—to air her grievances, and have them disposed of once and for all.

Whether that proposition commended itself or not to Miss Breay we are unable to affirm or deny. One thing, however, is quite clear, namely, that it was not agreeable to those who were really responsible for Miss Breay's resolution and the subsequent proceedings. Their sole desire appears to have been litigation, and accordingly Sir James Crichton Browne's proposal was rejected, and the action was commenced.

At the trial Mr. Commissioner Kerr seems to have had considerable doubt as to whether Miss Breay had any cause of action at all, but, following the practice of the judges of the High Court—a practice which has of late years come into vogue for the purpose of avoiding a new trial, and so saving expense to the parties—he ultimately decided to allow the case to go to the jury, directing them that, inasmuch as no damage whatever had been proved, they could not, even if they came to the conclusion that Miss Breay's rights had been infringed, award her more than nominal damages.

The jury found a verdict for Miss Breay, and assessed the damages at a farthing, and on October 15th Mr. Commissioner Kerr, who had, upon the application of Sir James Crichton Browne's counsel, reserved his decision on the points of law submitted to him, ordered judgment to be entered for Miss Breay in accordance with the findings of the jury, at the same time giving Sir James Crichton Browne leave to take the case to the Divisional Court. That appeal was heard on the 14th and 15th instant, when Mr. Justice Wills and Mr. Justice Wright set aside the verdict of the jury and the judgment of Mr. Commissioner Kerr, on the ground that there was absolutely no evidence fit to be submitted to any jury that Sir James Crichton Browne had acted otherwise than honestly and impartially, and also on the ground that Miss Breay's action was misconceived.

Miss Breay's counsel's application for leave to appeal was at once refused, Mr. Justice Wills saying that the proceedings were "very vexatious"; Mr. Justice Wright adding that "if the matter had been put into a statement of claim," that is to say, if the action had been originally brought in the High Court instead of in an inferior Court, he "would have struck it out as being frivolous and vexatious."

Now we disclaim the slightest intention of asserting, or even suggesting, that Miss Breay, or those associated with her in the above proceedings, were actuated in any step which they took by any desire to obtain any pecuniary benefit whatever from the transaction. It must be clearly understood that we make no such imputation. But, to some people, the uses of advertisement are sweet; it may be described as their life's blood, and as necessary for their existence as wholesome food and rest are to others. A craving for notoriety, to be, or be considered to be, fighting in the forefront of any battle, however unworthy the quarrel, is their one and only thought.

Unfortunately, however, this longing for advertisement not unfrequently leads to the wrecking of schemes such persons so loudly assert they are interested in, besides causing loss and annoyance to their neighbours. Such conduct cannot, and will not, be tolerated.

To the Executive of the Royal British Nurses' Association we have ever been prepared to accord our fullest sympathy. And, at no time more than the present, has that institution seemed to us to stand in need of all the sympathy it can obtain.

That a small clique of its members should be striving their utmost, by their conduct at its meetings, to bring it into ridicule and contempt, strikes us, we confess, as lamentable and shocking.

That for the purpose of accomplishing their ends they should incite and foster litigation, which has been described by those best qualified to form an opinion of its character as "frivolous and vexatious," may only be carrying their intentions to what they think is a logical conclusion, but it is none the less to be regretted.

We congratulate Sir James Crichton Browne upon his determination to obtain the judgment of the Divisional Court upon the matter; we congratulate him also on the result of his appeal.

We do not know whether Miss Breay will take the opportunity afforded to her of bringing her resolution before a meeting of the Association. If she and her supporters decide to do this, the smallness of her following can be made apparent. For the present, however, she and they have much to ponder over and consider. If the English language has any meaning, they have been publicly branded in open court as people who, with no other purpose to serve than that of harassing and annoying one whose only anxiety was to protect and serve them to the best of his skill and ability, have attempted to abuse the process of the law.

In the opinion of our contemporary, the *Nursing Record*, Miss Breay's action will "hereafter be regarded as an historical event in the nursing world." We agree. There are, alas! many events in history which have long been admitted on all hands to reflect little or no credit upon those who brought them about.

To that category in the history of the nursing world we confidently predict the conduct of Miss Breay, and her supporters will be relegated by all right-thinking people, and those who have the true interests of the nursing profession at heart.

